# An assessment of analytical performance using the six sigma scale in second-trimester maternal prenatal screening practices in China

**DOI:** 10.1016/j.plabm.2024.e00422

**Published:** 2024-07-23

**Authors:** Jinming Zhang, Xingtong Chen, Jiaming Wu, Penghui Feng, Wei Wang, Kun Zhong, Shuai Yuan, Yuxuan Du, Chuanbao Zhang, Falin He

**Affiliations:** a National Center for Clinical Laboratories, Institute of Geriatric Medicine, Chinese Academy of Medical Sciences, Beijing Hospital/ National Center of Gerontology, Beijing, PR China; b Chinese Academy of Medical Sciences and Peking Union Medical College, Beijing, PR China; c Department of Obstetrics and Gynecology, National Clinical Research Center for Obstetric & Gynecologic Diseases, Peking Union Medical College Hospital, Beijing, PR China

**Keywords:** Maternal serum screening tests, Quality control

## Abstract

**Objectives:**

We aimed to evaluate the analytical performance of second-trimester maternal serum screening in China, and to compare if there are differences in sigma levels across different methods and months.

**Methods:**

A retrospective study was conducted to assess the analytical quality levels of laboratories by calculating the Sigma metrics with prenatal screening biomarkers: AFP, Total β-hCG, free β-hCG, uE3. Data from 591 laboratories were selected. Sigma metrics were computed using the formula: Sigma metrics(σ) = (%TEa - |%Bias|)/%CV. The Friedman test and Mann-Whitney test were used to compare differences across various methods and different months. The Hodges–Lehmann was used for determining 95 % confidence intervals of pseudo-medians.

**Results:**

Only uE3 showed significant monthly variations in sigma calculations. However, around 8 % of laboratories across all four analytes demonstrated sigma levels both above 6 and below 3 in different months. Laboratories utilizing time-resolved fluorescence methods significantly outperformed those using chemiluminescence in sigma level. For AFP, the pseudo-median difference between these methods lies within a 95 % confidence interval of (−3.22, −1.93), while for uE3, it is at (−2.30, −1.40). Notably, the median sigma levels for all analytes reached the 4-sigma threshold, with free β-hCG even attaining the 6-sigma level.

**Conclusion:**

With current standards, China's second-trimester maternal serum screening is of relatively high analytical quality, and variations in sigma levels exist across different months and methods.

## 1 Introduction

Antenatal maternal serum screening assesses the risk of fetal chromosomal abnormalities (primarily trisomy 21), integrating specific biochemical markers with ultrasound imaging outcomes (such as nuchal translucency) and maternal factors (such as age and maternal weight) [[1], [2], [3], [4]]. Ever since Lo et al. [5] discovered cell-free fetal DNA, Non-Invasive Prenatal Testing (NIPT) has emerged as a hot topic in the field of prenatal screening, due to its relatively high positive predictive value [6] and comparatively low false-negative rate and false-positive rate [7,8]. According to health economics estimates, implementing Contingent Sequential Screening in China can maximize cost-effectiveness [9,10]. Moreover, certain underdeveloped regions lack access to modern molecular technologies, such as next-generation sequencing (NGS), necessary for NIPT implementation [11]. The complexity of ultrasound imaging programs [12] also results in variances in the uniformity and reliability of nuchal translucency (NT) in these areas. Consequently, maternal prenatal screening still plays a significant role in prenatal screening.

The quality control of maternal prenatal screening is an important guarantee of the reliability of prenatal screening. An analytical bias of 10 % in a single marker can alter the false positive rates by up to 2 % [13]. Considering China's 2022 birth population of about 9.65 million and a prenatal screening participation rate of 88.7 %, it is estimated that at least approximately 170,000 fetuses could be erroneously identified as positive in the initial screening. Although there is a lack of research specifically focusing on the CV's impact on maternal prenatal screening, study on other analyte suggest that the influence of CV is greater than that of bias [14,15].

Designed to boost quality, reduce costs, and foster continuous improvement, Sigma metrics have been used in clinical chemistry for over 20 years [16]. However, their application in prenatal screening remains rare. ‘Six Sigma’ refers to a statistical principle aiming to achieve a maximum of 3.4 defects per million opportunities (DPMO) [17,18]. It is generally accepted that the three-sigma level represents the minimum acceptable quality, and the six-sigma level signifies “world-class” quality [[19], [20], [21]]. James Westgard subsequently devised a formula for Sigma calculation [19], defined as Sigma metric(σ) = (allowable total error (TEa) (%) – |Bias (%)|)/(coefficient of variation (CV)). This formula aligns well with the analytical quality requirements of mid-trimester prenatal screening and Parvin's risk-based quality control model is a descendant of it. This study aims to introduce the concept of Six Sigma metrics into prenatal screening, assess the analytical quality of prenatal serum screening in China and compare Sigma levels across different months and methods.

## 2 Materials and methods

### 2.1 Inclusion and exclusion criteria

All participating laboratories in this survey are from ⅡA hospitals or above (IIIA > IIIB > IIA > IIB > I), and are in 31 provinces, autonomous regions, and municipalities throughout China. This research was centered on four analytes: Alpha-fetoprotein (AFP), Total beta-human chorionic gonadotropin (Total β-hCG), and Free beta-human chorionic gonadotropin (free β-hCG), along with Unconjugated Estriol (uE3). Participating laboratories were part of the National Center for Clinical Laboratories (NCCL) External Quality Assessment (EQA) Programs and submitted all internal quality control data for these biomarkers in February, July, and September of 2022. Given the difference in the concentration levels of quality control materials used by laboratories, laboratories that utilize two concentration levels of quality control materials were selected for sigma comparison. Any IQC results breaching quality control regulations were omitted to negate error-related impacts. In the end, the study incorporated data from 591 laboratories, including 528 laboratories for AFP, 243 for Total β-hCG, and 208 for free β-hCG, 406 for uE3.

### 2.2 Sigma metrics calculation

The Milan Conference on Global Analytical Quality Specifications identified state-of-the-art technology as the Model 3 for establishing Analytical Performance Specifications [22]. Due to the unavailability of other models, we use a TEa of 25 % based on the overall level of screening laboratories in China.

Bias was determined using the regression equation, constructed as y = b + ax, where x represents the robust average of peer laboratories, and y is the specific test value of a given laboratory. The Bias (%) was estimated by calculating the difference between the a and 1. When comparing monthly variations in sigma levels, bias was calculated using single-month EQA data (5 samples), while comparing sigma across different testing methods and calculating overall sigma levels, bias was calculated using three months of EQA data (15 samples).

The CV was obtained from IQC results submitted concurrently with the EQA data. Laboratories using quality control materials at a single concentration level constitute the majority, hence we selected only those laboratories.

When comparing monthly variations in sigma levels, CV was calculated using single-month IQC data, while comparing sigma across different testing methods and calculating overall sigma levels, bias was calculated using three months of IQC data.

**Fig. 1:**
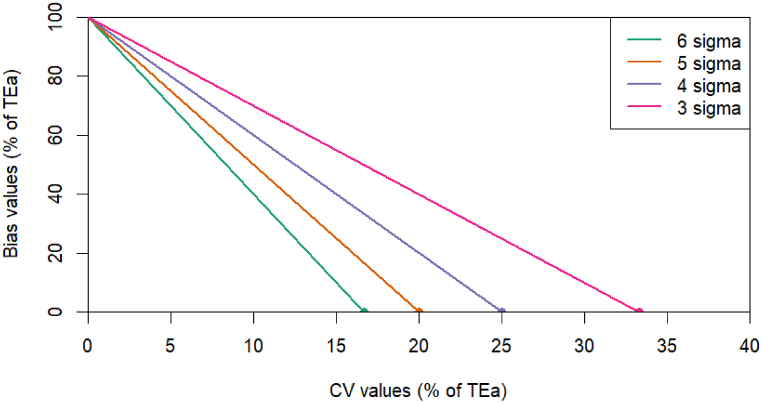
From left to right, the lines are for 6, 5, 4, and 3 sigma, respectively. The x-axis intersections are (16.67, 0), (20, 0), (25, 0), and (33.33, 0), and the y-axis intersection is (0, 10).

The Sigma metrics can be calculated from defined allowable TEa%, Bias%, CV% [23]:

$$\text{Sigma}\;\text{metrics}=\frac{\text{TEa}\%–\left|\text{Bias}\%\right|}{\text{CV}\%}$$

laboratories interested in using different TEa values can make approximate calculations based on Fig. 1.

### 2.3 Statistical analysis

To eliminate errors in reporting, Tukey's Test is used to exclude outliers. With Sigma metrics, a normal distribution is not expected. Therefore, the Friedman test for paired data sets of three groups. The Mann-Whitney test was employed for the analysis of two independent data sets, complemented by the Hodges–Lehmann estimation for determining 95 % confidence intervals of pseudo-medians. *P* < 0.05 was used as a significance level. All statistical analyses were performed in R language (R Foundation for Statistical Computing, Vienna, Austria), utilizing its inherent functions.

## 3 Results

### 3.1 Compare the sigma metrics for different months

**Fig. 2:**
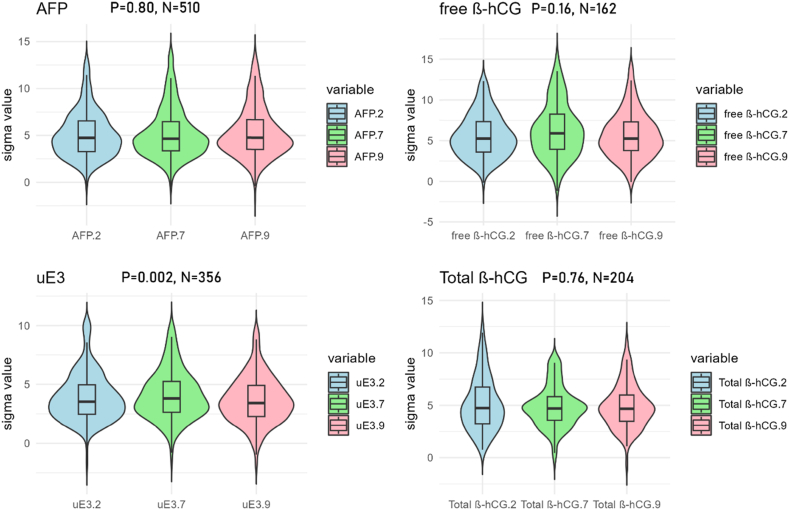
Monthly Comparison of Sigma Levels in Mid-Trimester Maternal Serum Screening laboratories in China. The “P" value in the graph represents the p-value of the Friedman test, and the “N" indicates the number of laboratories. From left to right, the graphs sequentially display the distribution of Sigma levels for February, July, and September.

The overall distribution of sigma levels for each month can be seen in Fig. 2. Statistical results are presented in Table 1. Only uE3 showed significant differences (*P* = 0.002, Friedman test). For all four analytes, approximately 8 % of laboratories displayed sigma levels both greater than 6 and less than 3 in different months (Table 1).

**Table 1:** Monthly sigma level and comparative analysis.

Analyte month	number	median (IQR)	P (Friedman)	proportion
**Alpha-fetoprotein**				
February	510	4.73(3.27)	0.80	8.4 % (43/510)
July		4.64(3.10)		
September		4.76(3.17)		
**Free beta-human chorionic gonadotropin**				
February	162	5.26(3.73)	0.16	8.0 % (13/162)
July		5.90(4.32)		
September		5.26(3.52)		
**Unconjugated Estriol**				
February	356	3.54(2.49)	0.002	8.4 % (30/356)
July		3.82(2.61)		
September		3.43(2.63)		
**Total beta-human chorionic gonadotropin**				
February	204	4.74(3.49)	0.76	6.9 % (14/204)
July		4.70(2.24)		
September		4.68(2.50)		

* median (IQR) refers to the median (IQR) of sigma, The proportion indicates the simultaneous occurrence of sigma levels above 6 and below 3 in three months.

### 3.2 Analysis quality indicators for major methods and systems

**Fig. 3:**
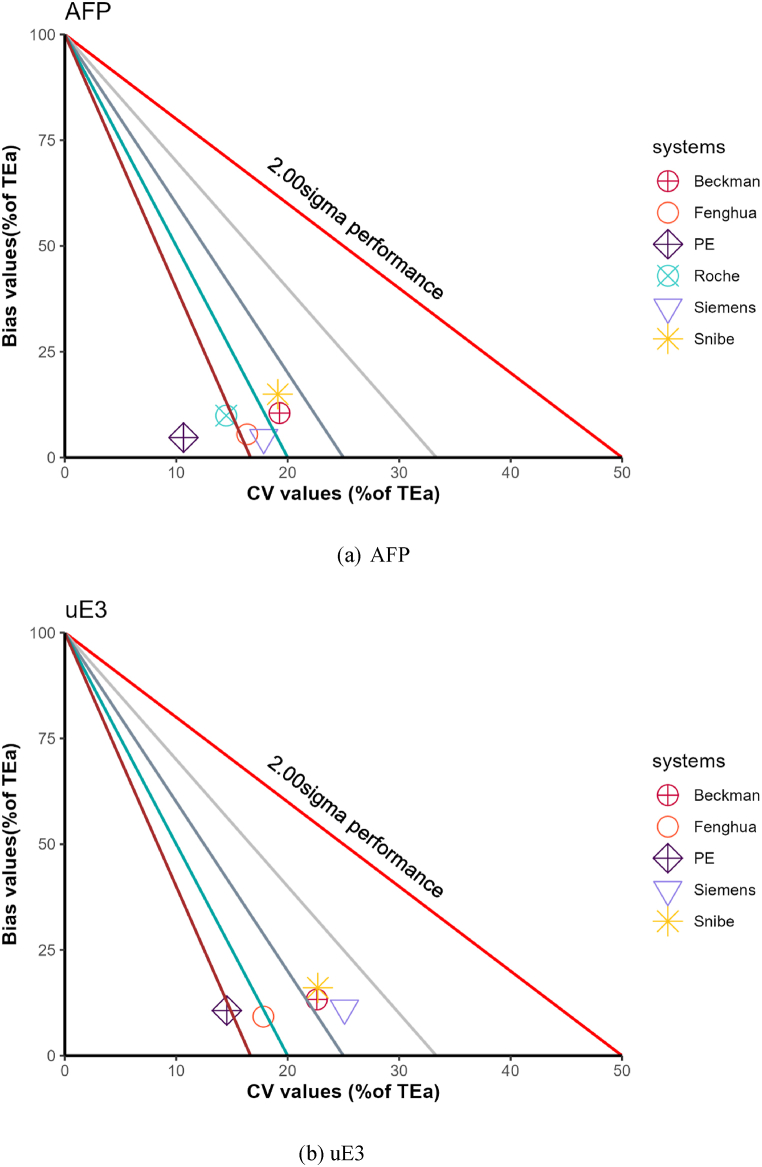
Normalized Sigma Method Decision Charts. CV and Bias have been normalized based on TEa, the x-axis in the graph represents the median CV of the system, while the y-axis represents the median bias of the system. The 2, 3, 4, 5, and 6 sigma performance lines are arranged from right to left.

PE, Fenghua employ time-resolved fluorescence (TRFIA), whereas Beckman, Siemens, Roche, Snibe utilize chemiluminescence (CL). The analytical quality levels for those methods and systems are presented in Table 2 and Fig. 3. The Mann-Whitney test indicated significant differences between the different methods in AFP and uE3 (Both p < 0.001). The 95 % confidence intervals for the pseudo-median differences (CL minus TRFIA) in AFP are (−3.22, −1.93), and for uE3, the intervals are (−2.30, −1.40).

**Table 2:** The |Bias|, CV, and sigma distribution of major methods and systems.

Table 2A. AFP										
Methods systems*	N	|Bias|			CV			Sigma		
		25th	50th	75th	25th	50th	75th	25th	50th	75th
**TRFIA**	**157**	**0.49**	**1.22**	**2.93**	**1.87**	**3.21**	**4.36**	**5.20**	**7.38**	**11.43**
PE	110	0.54	1.18	2.40	1.70	2.66	3.58	6.12	8.91	13.43
Fenghua	47	0.44	1.37	3.69	3.51	4.09	5.03	4.72	5.44	6.26
**CL**	**371**	**1.01**	**2.45**	**4.36**	**3.60**	**4.47**	**5.72**	**3.72**	**4.74**	**6.26**
Beckman	226	1.08	2.62	4.26	3.85	4.82	6.09	3.62	4.56	5.87
Siemens	43	0.74	1.17	2.18	3.82	4.46	5.43	4.39	5.35	6.00
Roche	67	1.13	2.48	5.10	2.66	3.62	4.72	4.53	6.02	8.43
Snibe	35	1.40	3.74	5.89	3.85	4.78	5.69	3.69	4.28	5.67

Table 2B free β-hCG										
Methods systems*	N	|Bias|			CV			Sigma		
		25th	50th	75th	25th	50th	75th	25th	50th	75th
**TRFIA**	**155**	**0.61**	**1.27**	**2.54**	**2.47**	**3.37**	**4.64**	**4.80**	**7.09**	**9.44**
PE	84	0.42	1.10	2.16	2.26	2.89	3.65	6.48	8.14	10.65
Fenghua	43	0.70	1.98	4.53	3.86	4.67	5.73	3.77	4.76	5.58

Table 2C uE3										
Methods systems*	N	|Bias|			CV			Sigma		
		25th	50th	75th	25th	50th	75th	25th	50th	75th
**TRFIA**	**127**	**1.09**	**2.52**	**4.48**	**3.04**	**3.78**	**5.00**	**4.21**	**5.45**	**7.08**
PE	84	1.21	2.67	4.31	2.91	3.63	4.65	4.41	6.01	7.57
Fenghua	43	0.65	2.31	4.69	3.54	4.45	5.46	3.90	5.16	6.61
**CL**	**275**	**1.61**	**3.32**	**5.52**	**4.51**	**5.69**	**7.07**	**2.78**	**3.70**	**4.89**
Beckman	207	1.50	3.32	5.49	4.51	5.65	7.07	2.80	3.77	4.90
Siemens	42	0.95	2.80	4.42	4.28	6.28	7.55	2.70	3.56	4.84
Snibe	26	2.91	4.01	8.96	4.97	5.68	6.31	2.91	4.01	8.96

Table 2D. Total β-hCG										
Methods systems*	N	|Bias|			CV			Sigma		
		25th	50th	75th	25th	50th	75th	25th	50th	75th
**CL**	**227**	**1.17**	**2.58**	**4.73**	**3.33**	**4.20**	**5.27**	**4.03**	**5.17**	**6.58**
Beckman	184	1.18	2.65	4.80	3.44	4.25	5.34	3.92	5.10	6.26
Roche	43	1.16	2.35	4.40	2.41	3.50	4.83	4.46	6.29	9.03

### 3.3 The overall sigma levels of biomarkers

**Fig. 4:**
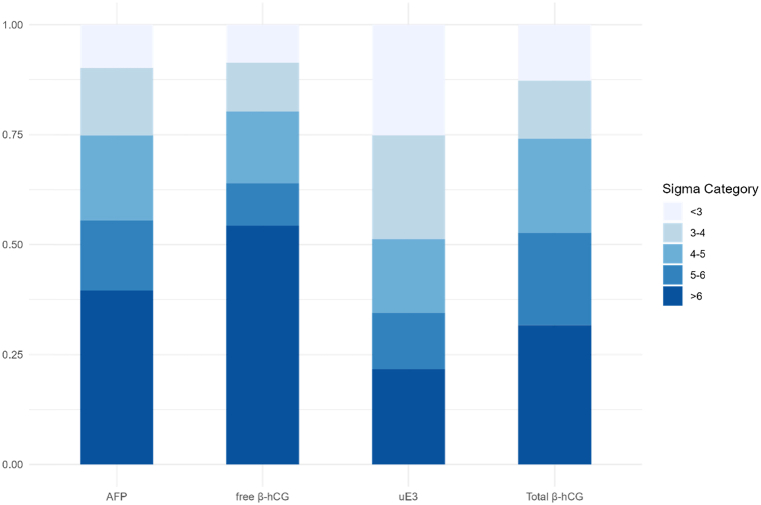
The sigma categorization of four analytes.

The median sigma levels for AFP, free β-hCG, Total β-hCG, and uE3 are 5.28, 6.38, 5.10 and 4.05, respectively. To better understand the distribution of sigma levels among various laboratories, the sigma levels of the five biomarkers were categorized into distinct intervals (<3 sigma; 3–4 sigma; 4–5 sigma; 5–6 sigma; ≥6 sigma), and the proportion of each group is presented in Table 3 and Fig. 4. The results indicate that the highest proportion of laboratories achieving a 6-sigma level was found in free β-hCG, at 54.3 %, while the highest proportion of laboratories below 3-sigma was observed in uE3, at 25.1 %.

**Table 3:** Sigma level grouping of 4 biomarkers.

	AFP	free β-hCG	uE3	Total β-hCG
<3sigma	9.8 % (52/528)	8.7 % (18/208)	25.1 % (102/406)	12.8 % (31/243)
3-4sigma	15.3 % (81/528)	11.1 % (23/208)	23.6 % (96/406)	13.2 % (32/243)
4-5sigma	19.3 % (102/528)	16.3 % (34/208)	16.7 % (68/406)	21.4 % (52/243)
5-6sigma	15.9 % (84/528)	9.6 % (20/208)	12.8 % (52/406)	21.0 % (51/243)
>6sigma	39.6 % (209/528)	54.3 % (113/208)	21.7 % (88/406)	31.6 % (77/243)

## 4 Discussion

This study introduces the concept of six sigma into the realm of laboratory prenatal screening analytical quality management, offering data from 591 Chinese laboratories as a reference for prenatal screening facilities. Researchers like Martín Yago et al. [24], James Westgard et al. [25], and Hassan Bayat [26] have simplified Parvin's risk management model and provided visualized charts to assist laboratories in selecting quality control rules and frequencies based on their own sigma levels, effectively reducing the probability of patient risks caused by error reports. For processes achieving Sigma metrics of 5.0 or higher, statistical quality control (SQC) designs are feasible. 4-Sigma processes demand increased diligence: they necessitate a higher number of control results for SQC procedures, the implementation of multiple rules, and result in reduced run sizes. Processes with Sigma metrics of 3.5 or lower will require even more sophisticated and expensive SQC approaches [25]. In fact, their focus should not be on designing internal quality control strategies, but rather on alternative control mechanisms [27]. The specific quality control rules and frequency selection corresponding to different sigma levels can be referenced from Westgard's articles [25].

Numerous studies have documented the time-dependent variability of sigma levels in laboratories [15,28,29]. The advantage of this study lies in its analysis of comprehensive data collected over different months from a wide array of laboratories. Although only uE3 showed statistical differences, approximately 8 % of laboratories for all four analytes exhibited sigma levels both greater than 6 and less than 3 across different months. Such marked variations suggest that analyzing sigma levels based on data from a single month may not provide a reliable assessment for prenatal screening laboratories. As depicted in Fig. 2, the overall distribution of sigma levels across different months appears relatively consistent. Hence, these disparities may stem from individual variability within the laboratories. Furthermore, sample size may also be a factor that makes it difficult to accurately estimate the CV. In China, prenatal screening laboratories typically perform tests once or twice a week, resulting in less frequent IQC. It is generally considered that estimating intermediate precision using data from 3 to 6 months is a reasonably suitable choice. Therefore, relying exclusively on one month's IQC data may not accurately estimate CV. Similarly, with only five instances of EQA, the reliability of bias calculations becomes equally questionable. These results are highly likely to be influenced by random errors.

Our findings indicate that, in terms of testing methods, laboratories using time-resolved fluorescence for AFP and uE3 significantly outperform those utilizing chemiluminescence. Specifically, when examining testing systems, the PE system (time-resolved fluorescence) exhibits outstanding performance, with Roche (chemiluminescence) and Fenghua (time-resolved fluorescence) also showing commendable results. This provides valuable insights for enhancing laboratory quality control measures. Due to the use of a competition-based assay for uE3, laboratories exhibiting performance below 3 sigma are more prevalent compared to other analytes. Additionally, the median sigma value is the lowest among them, recorded at 4.05. However, laboratories utilizing time-resolved fluorescence for uE3 report a median sigma level of 5.45, indicating a relatively favorable outcome. Laboratories aiming to elevate their quality control standards may find this information useful as a reference when considering system upgrades. Some may question the reliability of these outcomes. Due to the absence of reference methods, target values derived from the robust mean of peer laboratories were used. This suggests that bias is more indicative of a laboratory's standing among its peers. Nevertheless, this also demonstrates that methods or systems with minimal bias yield more comparable results, facilitating the mutual recognition of test outcomes. Moreover, evidence from the literature indicates that CV exerts a more significant influence on sigma levels than bias [14,15]. Certain studies propose that bias should be excluded from sigma calculations [30,31], and the CLSI C24-Ed4 document also considers it as an option [32]. In this situation, even when considering solely the perspective of CV, laboratories using time-resolved fluorescence experiments are still superior to those using chemiluminescence.

TEa may represent the most significant indicator influencing sigma levels [15]. The Milan strategic conference [22] proposed three models for deriving Analytical Performance Specifications based on [1] clinical outcomes [2], biological variation, and [3] state-of-the-art technology. The multiplicity of models for TEa often presents a challenge in selection [33]. However, for the four prenatal screening analytes discussed in this paper, models based on clinical outcomes and biological variation are notably absent. We have selected a TEa standard of 25 % in China. Considering the semi-punitive nature of China's EQA program, it is important that the TEa is set at a level that allows 80 % of laboratories to pass the EQA. Although it may be applicable to tumor markers, AFP has a CLIA 2024 goal of 20 %, a desirable Ricos goal of 21.9 %, and an EFLM minimum goal of 26.5 %. Meanwhile, beta hCG has CLIA 2024 goals of 18 % or 3 mIU/mL. From this perspective, our TEa choice is still relatively reasonable. However, our analysis indicates that the four analytes, which exhibit significantly different sigma level distributions, are not suitable for the same TEa standards. We recommend that relevant authorities establish more rational and scientific TEa values, tailored to the characteristics of each analyte, to ensure patient welfare.

A notable limitation of this study is its exclusive inclusion of laboratories that use quality control materials at a single concentration level, despite these laboratories representing the largest proportion. The CV often differs across different concentration levels of quality control materials, with higher concentration controls typically exhibiting lower CVs. This poses a challenge in calculating sigma levels. For laboratories where CVs remain consistent across various concentration levels, averaging the CVs or sigma levels seems reasonable. However, for those with substantial CV differences, the choice becomes more complex. Some suggest using the lower sigma levels [[34], [35], [36]], while others believe in averaging the sigma levels, but to maintain balance, sigma values greater than 6 should be considered as 6 sigma [37]. These approaches may be feasible for individual laboratories but pose challenges for our statistical analysis. Therefore, we only included laboratories using quality control materials at a single concentration level. Nonetheless, from a naive perspective, laboratories employing multiple concentration levels of quality control materials likely place a higher emphasis on analytical quality control and could be expected to achieve comparable or higher sigma levels.

From the perspective of internal quality control, the overall analytical performance of the four mid-trimester serum markers for prenatal screening is quite good. Compared to the lower sigma levels required for glycated hemoglobin, which generally only needs to reach 2 sigma, even the median sigma level for uE3 is at 4.05. This allows laboratories to achieve higher sigma levels more easily, which in turn means that they can adopt more lenient quality control rules and lower frequencies of quality control. However, we have focused solely on the analytical performance of mid-trimester serum markers in Chinese laboratories. Further analysis is needed to assess the screening performance of these markers, which requires studying indicators such as MoM values, detection rates, and positive predictive values. This will be the focus of our future research direction.

## Ethical approval

Not applicable.

## Research funding

This work was supported by grants from the National Key Research and Development Program of China(2021YFC1005304, 2022YFC3602301,2022YFF0710305).

## CRediT authorship contribution statement

**Jinming Zhang:** Writing – review & editing, Writing – original draft, Visualization, Validation, Supervision, Software, Resources, Project administration, Methodology, Investigation, Formal analysis, Data curation, Conceptualization. **Xingtong Chen:** Writing – review & editing, Writing – original draft, Conceptualization. **Jiaming Wu:** Data curation, Formal analysis, Software, Supervision, Validation, Visualization, Writing – review & editing. **Penghui Feng:** Writing – review & editing, Data curation, Conceptualization. **Wei Wang:** Writing – review & editing, Writing – original draft. **Kun Zhong:** Writing – review & editing, Writing – original draft. **Shuai Yuan:** Writing – review & editing, Writing – original draft. **Yuxuan Du:** Writing – review & editing, Writing – original draft. **Chuanbao Zhang:** Writing – review & editing, Writing – original draft, Visualization, Validation, Supervision, Software, Resources, Project administration, Methodology, Investigation, Funding acquisition, Formal analysis, Data curation, Conceptualization. **Falin He:** Writing – review & editing, Writing – original draft, Visualization, Validation, Supervision, Software, Resources, Project administration, Methodology, Investigation, Formal analysis, Data curation, Conceptualization.

## Declaration of competing interest

The authors declare the following financial interests/personal relationships which may be considered as potential competing interests:

Chuanbao Zhang and Falin He reports financial support was provided by 10.13039/501100013290National Key Research and Development Program of China. If there are other authors, they declare that they have no known competing financial interests or personal relationships that could have appeared to influence the work reported in this paper.

## Data Availability

Data will be made available on request.
